# A qualitative exploration of perceptions and experiences of contraceptive use, abortion and post-abortion family planning services (PAFP) in three provinces in China

**DOI:** 10.1186/s12905-017-0458-z

**Published:** 2017-11-21

**Authors:** Yan Che, Esther Dusabe-Richards, Shangchun Wu, Yi Jiang, Xiaojing Dong, Jian Li, Wei-Hong Zhang, Marleen Temmerman, Rachel Tolhurst, INPAC group, Marleen Temmerman, Wei-Hong Zhang, Longmei Tang, Su Zhou, Jialin Xu, Yip Ching Yu, Jian Li, Chengliang Xiong, Junli Liu, Qinglong Meng, Yan Che, Weili Zhao, Huiping Zhang, Xu Qian, Hong Jiang, Ji Liang, Shangchun Wu, Yan Zhou, Qing Liu, Lina Hu, Xiaojing Dong, Yi Jiang, Shuaibin Liu, Xiaoling Gan, Jørn Olsen, Jiong Li, Rachel Tolhurst, Dusabe-Richards Esther

**Affiliations:** 1 Key Laboratory of Reproduction Regulation of NPFPC (SIPPR, IRD), Fudan University, Shanghai, China; 2 Chinese Society for Family Planning, China Medical Association, Beijing, China; 3 Department of International Public Health, Liverpool School of Tropical Medicine, Pembroke Place, Liverpool, L3 5QA UK; 4 National Research Institute for Family Planning, Beijing, China; 5 Public Health and Management Department, Chongqing Medical University, Chongqing, China; 6 The second affiliated hospital of Chongqing Medical University, Chongqing, China; 7 International Centre for Reproductive Health, University of Ghent, Ghent, Belgium

**Keywords:** Abortion, Post-abortion family planning (PAFP), Contraception, China

## Abstract

**Background:**

The INPAC project aims to evaluate the effectiveness of integrated post-abortion family planning (PAFP) services into existing hospital based abortion services in China. A qualitative study was conducted in three provinces to contribute to developing effective PAFP services through understanding influences on contraceptive use, experiences of abortion and existing PAFP, and their effect on future contraceptive practices from the perspective of users, in the context of social and institutional change.

**Methods:**

Twenty-nine in-depth interviews (IDIs) were undertaken with women who had experienced abortion between 1 and 6 months prior to interview, recruited from three urban and two rural facilities in each province. Thirteen IDIs were also conducted with male partners. Six focus group discussions (FGDs) were carried out with community members from different social groups, including unmarried and married women and men, urban residents and rural-to-urban migrants.

**Results:**

Social networks and norms are important in shaping attitudes and behaviour towards abortion and contraception. Widespread concerns were expressed about side-effects, reliability and effects on future fertility of some modern contraceptives. The combination of limited information and choices and a lack of person-centred counselling in PAFP with anxieties about side effects underlies the widespread use of unreliable methods. Gendered power relations significantly influence contraceptive (non)use, with several examples illustrating women’s relative lack of power to decide on a method, particularly in the case of condoms. Although the availability of contraceptive information from respected providers can offer impetus for individual behaviour change, social distance from providers reduces opportunities for clients to discuss their difficulties regarding contraceptive use; particularly, but not exclusively for young, unmarried clients.

**Conclusions:**

Increased access to non-commercial, reliable information on contraceptive methods is needed. PAFP services must go beyond simple information provision to ensure that providers take a more person-centred approach, which considers the most appropriate method for individual clients and probes for the underlying influences on contraceptive (non)use. More sensitive reflection on gender norms and relationships is required during counselling and, where women choose this, efforts should be made to include their male partners. Specific attention to provider positionality and skills for counselling young, unmarried clients is needed.

## Background

Sexual and reproductive rights are integral to the health and wellbeing of women and girls across the lifecycle. Principal among these rights is the recognition that women should have the available means and agency to decide whether, when and how to bear children [1]. While access to all forms of contraception and access to safe abortion represent critical facilitators of this right; in many parts of globe there remains substantial unmet need for contraception as well as considerable legal barriers to safe abortion [2].

There is limited evidence on the long-term health consequences of induced abortion. Safe abortion poses low risks to a woman’s health, especially in comparison with the risks posed by pregnancy, especially when unintended. However, there is some evidence that induced abortion and particularly surgical abortion, increases the risk of complications and poor outcomes in future pregnancies, including low birthweight and preterm birth [3–6]. There is weaker evidence for increased risk of mood disorders [5, 7]. There is also evidence that the risks of low birthweight and pre-term births increase with subsequent abortions [6]. This suggests that whilst access to safe abortion is a critical reproductive right, preventing unintended pregnancy where possible through access to high quality contraceptive services is also important to promote women’s health, particularly in future pregnancies.

Induced abortion has been legal in China since the 1950s. Around half of unintended pregnancies are attributed to contraceptive failure (40–56%) and the other half to non-use of contraceptives (44–50%) [8, 9], raising questions around influences on utilisation of effective contraception.

The Chinese government has implemented a strict family planning (FP) policy framework to attempt to control population growth since 1979, although this has undergone significant reforms over the past three and a half decades [10]. Current policy has shifted away from mandatory insertion of Intra-Uterine Devices (IUDs) for women with one child, abortion for ‘unauthorized’ pregnancies, and sterilization for couples with two or more children, towards a more client-centred approach. The new approach offers informed choice on contraception, rights to decide on the spacing and timing of a (locally determined) limited number of ‘authorised’ births, and the allowance of ‘compensation fees’ to enable unauthorised pregnancies to proceed [10]. However, institutional norms and practices often lag behind changed policy [10].

China’s total contraceptive prevalence rates remain at the highest level in the world at 89.20% among married women in China 2010 [11]. Long-term contraceptive usage is especially high: among married women of reproductive age in 2010 intrauterine device usage stood at 48.15% and female sterilisation at 31.7% [10]. In contrast, usage of oral contraception and condoms is relatively low (at 0.98% and 9.32% respectively in 2010) [10]. Key factors influencing this include the history of mandatory long-term contraception, relatively low awareness and information about short-term methods such as oral contraceptives, related misconceptions about oral contraceptive side effects and deficiencies in the provision system for oral contraceptives, which until recently were available at no cost at family planning clinics but not at public hospitals [12].

Until 2013 FP policy was implemented by the Family Planning Commission, which controlled the authorisation of births and provided family planning services to married couples only. Although Family Planning clinics provide abortion services, the majority of abortions are performed in public hospitals, which have been managed by a separate Ministry of Health. Partly as a result of this separation, post-abortion contraceptive counselling and services have rarely been provided to women following an abortion, nor are women usually referred to family planning clinics [8]. In 2013 the Family Planning Commission merged with the Ministry of Health at national level. Although the implementation of the merger is incomplete at the level of service delivery, it offers opportunities to integrate contraceptive/FP counselling and services into abortion service provision with the aim of reducing subsequent abortions.

Rapid economic and social change since the 1980s has driven shifts in sexual attitudes and behaviour. Despite the continued focus of official FP policy on married couples, recent reviews of the literature suggest that there is an increasing acceptance of premarital sex and extramarital sex in China, especially among young people and in urban areas [13–15]. The official age of marriage in China is 20 for women and 22 for men. The prevalence of regular contraceptive use among unmarried women varies widely by year between 17 and 70% without showing an overall increase (Li et al., 2013). A substantial number of pregnancies among unmarried women in China result in induced abortion [8, 16, 17].

A survey of women service users under the age of 25 conducted in three urban abortion clinics in China found that the rate of repeat abortion was 35.0% and that of those using the services, 75.1% were unmarried (although more than half cohabited with a partner), more than half were migrants and of those younger than 20 (14.1%) 20% did not have a high school education [8]. A survey among over 18,000 women undergoing induced abortion due to unintended pregnancy in Beijing in 2010–11 found that migrants accounted for the majority of the abortion service users [18]. Cross-sectional studies focused on rural-to-urban migrants have identified high rates of abortion among these groups, especially among those who are young and unmarried, as well as general lack of knowledge about sexual and reproductive health issues [17, 19–21].

In response to those concerns, the four-phases collaborative research project funded by European Commission (EC) under the Seventh Framework Programme (FP7) on INtegrating Post-Abortion family planning services into existing abortion services in hospital settings in China (INPAC) is being undertaken.^1^ The INPAC project aims: first, to evaluate the effectiveness of integrated post-abortion family planning (PAFP) services into existing hospital based abortion services in China on the reduction of unintended pregnancies and subsequent abortions; and second to assess the practicability of integrating PAFP into existing abortion services in participating hospitals through a health system study involving stakeholders: policy makers, health managers, abortion service providers and women who have undergone abortion.

The first phase of this project aimed to inform the development of the INPAC intervention (second phase) by undertaking a mixed method situation analysis of the current health system and social context for integrating PAFP into existing abortion services. Developing effective PAFP services requires an understanding of influences on contraceptive decision making, experiences of abortion and PAFP and their influence on future contraceptive decision-making from the perspective of users and within their social context. Very few qualitative studies of contraceptive decision-making have been conducted in China and we could not identify any qualitative studies of user experiences of abortion, PAFP and post abortion contraceptive decisions. This paper reports qualitative results from in-depth interviews with abortion service users and their male partners, and focus group discussions with specific groups of potential service users (adults within the general population). The interviews explored perceptions and decision making around contraceptive use, experiences of abortion services, including PAFP services, and post-abortion contraceptive decision-making.

## Methods

### Study sites

This study was carried out in three provinces of China in 2013. These three provinces represented different areas of socio-economic development within the country: Zhejiang, a highly developed province located in the eastern coastal area, had a GDP per capita of 68,593Yuan (about 11,075 US$) in 2013. Hubei, with a medium level of development, located in central area of China had a GDP per capita of 42,686 Yuan (about 6892 US$) in 2013. Yunnan, a relatively poor and undeveloped province, had a GDP per capita of 25,157 Yuan (about 4062 US$) in 2013 and 33% of its population are ethnic minority groups. Within each province, one city and one rural county were selected, with representation across the sample of: relatively rich and poor cities/counties; ethnic minorities (one county); rural-to-urban migrants (one city). In each city, three facilities were selected for participant recruitment: one level-2 hospital (medium sized hospital) and two level-3 hospitals (one general or comprehensive hospital at city level and one maternal and child health hospital at city level). In each rural county, two facilities were selected: one level-1 hospital (township hospital), and one level-2 hospital (county level). In Zhejiang province, in the rural setting, one Maternal and Child Health institute and one level-2 hospital were selected as these two institutions provide the majority of abortion services in the area. We recruited service users and their partners for in-depth interviews via facility records.

### Data collection

Twenty-nine in-depth interviews (IDIs) were undertaken with women who had experienced abortion between 1 and 6 months prior to the interview. A purposive sample of women was selected at different levels of hospitals by using the abortion register to include maximum variation amongst interviewees. Factors taken into consideration were age, marital status, and residency status (migrant or permanent urban resident (see Table 1 for participant characteristics). Interviews sought to explore, from the women’s perspectives, the circumstances of the abortion, experiences of care received, future reproductive and contraceptive plans and influences on these, and perceptions of the acceptability of planned PAFP services. Thirteen in-depth interviews were also undertaken with male partners of a sub-sample of these women. These men were invited for interview following an informed consent process with their partners who gave permission for the research team to request an interview. It was important to include these men, since literature suggests they may be influential on contraceptive uptake [22] (see Table 2 for breakdown of numbers).

**Table 1:** Socio-demographic characteristics of women service users participating in individual in-depth interviews (IDIs) in Yunnan, Hubei and Zhejiang

	Marital status	Employment status	Education	Residency status	Number of abortions	Existing children
Yunnan province	Divorced	Worker	Technical secondary school	Permanent Country level	2	0
	Married	Farmer	Junior high School	Permanent Country level	1	1
	Unmarried	Unemployed	Junior high School	Migrant Country level	Number of abortions is unclear	0
	Married	Farmer	Senior high School	Permanent Country level	1	1
	Married	Farmer	Junior high School	Permanent Country level	1	0
	Married	Farmer	Junior high School	Permanent Country level	2	1
	Married	Farmer	Primary School	Permanent Country level	1	1
	Married	Farmer	Primary School	Permanent Country level	1	1
	Married	Worker	Primary School	Permanent Country level	1	1
	Married	Farmer	Illiterate	Permanent Country level	1	2
Hubei province	Not known	Public official	University	Urban districts in Suizhou	5	1
	Married	Company employee	High school	Rural districts in the rest of cities	1	1
	Married	Worker	High school	Rural districts in Suizhou	1	1
	Unmarried	Unemployed	High school	Urban districts in Suizhou	1	0
	Married	Company employee	University	Urban districts in Suizhou	1	1
	Married	Commercial people	High school	Urban districts in Suizhou	1	1
	Married	Unemployed	High school	Rural districts in Suizhou	1	1
	Married	Unemployed	University	Rural districts in the rest of cities	1	0
	Unmarried	Unemployed	Diploma	Urban districts in Suizhou	1	0
	Married	Public official	Diploma	Urban districts in Suizhou	1	1
	Married	Public official	University	Urban districts in Suizhou		1
Zhejiang province	Unmarried	Worker	Senior high school	Migrant country level	1	1
	Married	Worker	Junior high school	Migrant country level	1	1
	Unmarried	Teacher	Technical secondary school	Permanent city level	1	0
	Married	Independent business woman	University	Local resident	1	0
	Married	Unemployed	Senior high School	Migrant	2	1
	Married	Independent business woman	Primary school	Migrant	2	2
	Married	Unemployed	Junior high school	Local resident	3	1
	Married	Commercial service (hotel)	Junior high school	Migrant	1	1

**Table 2:** Male partners participating in individual in-depth interviews (IDIs) in Yunnan, Hubei and Zhejiang

Province	Area	Number of Participants
Yunnan	Urban	2
	Rural	2
Hubei	Urban	2
	Rural	2
Zhejiang	Urban	3
	Rural	2

To capture broader views on abortion, six focus group discussions (FGDs) were carried out with community members from different social groups: including unmarried and married women and men, urban residents and rural-to-urban migrants *(see* Table 3 *for breakdown of numbers).* These FGDs were used to explore the social context for FP and abortion, including: acceptability of pre-marital sex and pregnancy; perceptions, knowledge and attitudes towards FP and abortion; perceptions of abortion providers; desired sources and media for FP information; and perceptions of appropriateness. See Table 4 for a summary of data collection methods.

**Table 3:** Men and women participating in focus group discussions (FGDs) in Yunnan, Hubei and Zhejiang

Province	Participant category	Number of participants
Yunnan	Married Women (Dai ethnicity)	6
	Married Men (Dai ethnicity)	7
Hubei	Unmarried Women	6
	Unmarried Men	6
Zhejiang	Unmarried rural women	6
	Married rural women	6
Total		37

**Table 4:** Summary of method and participant type

Method and participant type	Study site level	Total number
IDIs with women who experienced abortion	Urban	13
	Rural	14
IDIs with male partners of women who have experienced abortion	Urban	7
	Rural	6
FGDs with different social groups(unmarried people (male/female), married ethnic minority (male/female), married migrant women and unmarried migrant women)	Urban or rural	7
Total sample	In-depth interviews	40
	FGDs	7

Semi-structured topic guides were used in interviews and FGDs. Three research teams were formed and each team consisted of one senior, one middle-level and one junior researcher. Topic guides were developed and discussed collaboratively among team members before being piloted and implemented. All interviews were tape-recorded with the consent of interviewees. All IDIs and FGDs were conducted in a private room to avoid interruption from outside. Each IDI lasted for approximately 30 to 40 min and Each FGD lasted for about 1 h. Each team checked the contents of respective interviews. To ensure the quality of the interviews, interviewers repeated the summary of interview to interviewees to check the answers after the interview was done. After the interview, a stenographer transcribed the whole interview verbatim based on recordings. Another team member double checked the text of each interview.

### Data analysis

All the recorded files were managed systematically by assigning a unique identification number to each interview so as to ensure the anonymity of the respondent. Data were analysed using a framework approach [23] as follows. Transcripts were read and re-read separately by the investigators to identify emerging themes. Data were coded according to predetermined analytical categories and organized into themes and subthemes. Summary matrices were developed for every category of respondents in each province by comparing, contrasting and synthesizing information within and across cases. The thematic framework was used to classify and organize data according to key themes and concepts categories.

### Ethical approval

Ethical approval was obtained from the Liverpool School of Tropical Medicine Research Ethics Committee, and the Institutional Review Boards of the School of Public Health, Fudan University, National Institute for Research in Family Planning, and the Chongqing Medicine University Ethics Committee, China. Oral, informed consent was obtained from all participants of IDIs and FGDs. All IDIs and FGDs were recorded with respondents’ consent.

## Results

We identified four main themes as follows: 1) Influences of the changing social environment on sexual behaviour and contraceptive use; 2) Fears about and experiences of negative health impacts of contraceptive methods influence contraceptive decisions; 3) Gendered power and communication in relationships influence contraceptive use and PAFP; and 4) Limited and directive counselling were common experiences of PAFP.

### Influences of changing social environment on sexual behaviour contraceptive use: Social norms, social networks and commercial interests

#### Changing social attitudes towards sex and abortion

The majority of participants across all provinces, in rural and urban areas, perceived that premarital sex has become increasingly common in China, where it had been previously perceived as ‘shameful’:

*Young people aged 18–22 have no stress at all in their own lives, especially for those born in 1990s.They feel empty* 2 *about their lives, and they’re happy with the premarital relationship. Their parents wouldn’t worry that much…Those young couples are happy being together. They never think about the future.*

(IDI with married migrant woman, Zhejiang, high GDP context).

However, embarrassment about discussing contraceptive use remains:

*There are no free condoms in my community. Well, I do not know if there are any. Even if there are any, it is too embarrassing to collect them.*

(IDI unmarried woman, Yunnan, low GDP context).

Premarital abortion also still carries a social stigma in current Chinese society. Young women in Hubei reported that they would not want parents to know about their premarital sexual behaviour and both women and men agreed that parents and society would generally be more lenient with sons rather than daughters. Hence some unmarried women tended to use private hospitals to access abortion services.

*Participant 1: It depends on which kind of person they are. If she’s a student at the age of 16–17, [having an abortion] will leave her with a bad reputation. But if she’s 23–24 years old, it doesn’t matter.*

*Participant 5: People will always judge the girls. A girl’s virginity has been very important ever since the ancient times, yet no boy has been blamed.*

(selected quotes from FGD with young women, Zhejiang, high GDP context).

#### Social norms and social network influences on perceptions about appropriate methods

In discussing the reasons for contraceptive choices, participants in FGDs and IDIs revealed a number of social norms and ‘common-sense’ perceptions around contraceptives and their use at particular stages of life – that is, before and after marriage and birth of a first child.

A common norm expressed in both interviews and FGDs was that use of IUDs is only appropriate following the birth of a first child and hence by married women. In one case a married woman in an IDI specifically stated that she had an IUD inserted after the birth of her first child due to local family planning policy. However, the majority simply stated this as ‘common-sense’ or referred to common practice around them. A minority referred to female sterilisation following a second child as common practice.

*P1: Usually women use IUD after childbirth.*

*P2: [After Childbirth, women can have] IUD or OCs. Anyway, they already had baby.*

*P3: It’s safe to use IUD after people have baby since it is effective.*

(Selected quotes from FGD with married migrant women, Zhejiang, high GDP context).

Decisions to use IUDs were influenced by individuals’ social networks, including women’s own mothers’ contraceptive use; one married interviewee referred to IUDs as a ‘method handed down from our parents’ generation’.^3^

Condoms were commonly seen a particularly appropriate method for unmarried women and men, although their use was also seen as an option for married couples, particularly before they had one child or if an IUD was ‘not suitable’ for the woman. In general (short-acting) oral contraceptives (OCs) were seen as a potential option prior to the birth of a first child, particularly for married couples, but the vast majority of participants across provinces, method and interviewee type held very negative views about their use (see theme 2).

Abstinence-based approaches, such as the ‘rhythm method’ or ‘safe period’ (a method based on abstinence from sex during fertile periods) were also perceived as commonly used methods, and many individual interviewees reported using these methods after hearing about them from friends. They were not necessarily perceived as effective, but often used as a default option in cases where the couple were not able to decide or agree on a modern method.

#### Media and commercial influences on perceptions

Participants in both FGDs and IDIs described the media as common sources of contraceptive information, primarily internet and television, including advertising. The internet was the most popular source of contraceptive information for married couples and unmarried men. However, some were wary about commercial sites, and said they would not necessarily trust them. A group of young unmarried men agreed that the promotion of so-called ‘painless’ abortion (carried out under general anaesthetic) by private-sector hospitals encouraged people to see abortion as an easy solution to unintended pregnancy:

*“Advertisements, particularly about painless abortion, showing on television, radio and billboards on cars are too many, which make people feel abortions are too easy and normal”.*

(FGD with unmarried men, Hubei, medium GDP context).

The influences of commercial interests and the commodification of health were visible in the sense of mistrust of free contraceptive products offered by government health services. For example, an unmarried man in Yunnan province reported during an FGD that he would be concerned about the quality of free condoms in comparison to bought condoms. He suggested that this was similar to buying peace of mind by paying for a parking space to protect one’s car:

*The quality of free condoms is somewhat poorer (than bought condoms). This is just like you would not be worried about someone throwing stones at your car if you parked it in a paid parking lot.*

(FGD with unmarried male partners, Yunnan, low GDP context).

A perceived hierarchy of quality in types of IUD also emerged, as exemplified by one rural woman explaining that she intended to travel to an urban centre to get a ‘better quality’ IUD or contraceptive implant inserted; she perceived the type of IUD available for free in her township health centre as poorer quality than one for which she would have to pay (200 yuan) at a higher level (Level 2 or above).

### Fears about and experiences of negative health impacts of contraceptive methods

Concerns about potential health risks associated with the use of specific contraceptives emerged as an important influence on contraceptive choices. Some participants drew on their own experiences (mainly negative), and others expressed perceptions based on the experiences of people they knew. Many, however, referred to common-sense notions, expressed as ‘just what I think’, or the social diffusion of knowledge (‘I have heard’). OCs in particular were commonly described as ‘harmful to health’, which was often explicitly related to their hormonal nature. Common specific harms perceived were irregular menstruation or bleeding, and future infertility or delayed fertility after discontinuing use. Less commonly mentioned fears included gaining weight and experiencing ‘stomach problems’. Few in either FGDs or interviews spoke from personal experience about OCs, and generally stated this as ‘common-sense’. Only one unmarried woman said she had heard OCs were not bad for health from her friends.

*I would not like to take OCs. There will be side effects. OCs are a kind of hormone. It will make my menstruation irregular.*

(IDI with woman service user, Zhejiang, high GDP context).

*My aunts scolded me (for using pills) after I got married… they told me that the pills were not good, which would cause infertility when you really want a child.*

(FGD with married migrant women, Zhejiang, high GDP context).

Concerns about IUDs centred on a perception of them as invasive to the body; they were referred to by more than one participant as ‘foreign matter’ and several women expressed the perception that the IUD might be ‘expelled’ from the body. These fears were prompted by both the experiences of others within their social network, and ‘common sense’ norms.

A number of women, both in individual interviews and FGDs, reported experiencing problems with the use of IUDs, including physical discomfort, abdominal pain, and menstrual/bleeding problems, which sometimes led to them having the device removed. Two women had experienced a pregnancy with the IUD in situ, one of which was ectopic. One woman had previously experienced cervicitis and therefore felt an IUD was not suitable for her.

Less clear norms emerged regarding condoms. Several married women in IDIs described a general feeling of condoms being ‘not good’, ‘not good for the womb’ or ‘not clean’ especially if used repeatedly or in the long term, but were not able to articulate any reasons for this. One married woman described condoms as ‘not safe’ (i.e. not effective). However, two women expressed positive feelings about condoms, saying that they were good and were ‘hygienic’.

*Interviewee: my husband is a caring man. He thought that taking pills might hurt my health. He would like to use condoms. But I know in my heart that condoms are not very good. It is not very good to use them often.*

*Interviewer: why do you think so?*

*Interviewee: I do not know what side effects [I] would have while using condoms. I just know they are not clean.*

(IDI with married woman, service user Hubei, medium GDP context).

The above quote illustrates how participants narrated weighing up the relative potential for harm from different methods, as well as the commonly held perception of oral contraceptives as especially harmful.

Some participants also expressed an awareness of potential negative impacts of using Emergency Contraceptives, which were considered to be a contraceptive method by some female respondents, particularly unmarried women:

*I do not know much about it. Anyhow, I just know eating emergency pills is not good. But I do not know its disadvantages. I really do not know.*

(IDI with unmarried woman, service user, Yunnan, low GDP context).

### Relationships: Gendered power and communication influence contraceptive use and PAFP

#### Gender relations influence decisions about contraceptive use

The majority of respondents across all provinces perceived that ideally men and women should both take responsibility for making decisions about contraception within a relationship. However, decision-making power was often unevenly distributed among couples, and decision-making patterns and power varied within and outside, or prior to, marriage. This unevenness manifested in a variety of ways.

A number of married female participants expressed that women were usually more concerned about contraception and would therefore take more responsibility, either after discussion with partner or independently.

*We usually discuss which method to use…[he] usually respects my opinion.*

(IDI with married woman service user, Hubei, medium GDP context).

However not all participants across the sample agreed with this, and a number of married women and men reported that husbands had primary influence over decisions on when and what types of contraception were used:

*I had chat with my husband on contraceptive use. He said drugs will damage my health…Women will be fat if they take OC. Drugs always have side effects. He said he was willing to use the condom… We do not use other methods.*

(IDI with married woman service user, Zhejiang, high GDP context).

A number of married and unmarried women in both interviews and FGDs cited the refusal of husbands and partners to use condoms, or to use them consistently, as a significant barrier to consistent use of this method. For example, one married woman in rural Yunnan said that she initially wanted to use either condoms or IUD but her husband refused. After the abortion he ‘agreed’ that she could have an IUD inserted since he was concerned that subsequent abortions would be bad for his wife’s health and he did not want to use condoms; she was clear that he made the final decision here. Others perceived men’s reluctance as a general problem or anticipated their own partner’s dislike of the method. One married woman described her husband’s willingness to use the method as evidence of his ‘caring’ behaviour.

In Hubei unmarried women (in a FGD) discussed a range of ways in which their partners influenced decisions on their use of contraceptives: one depended on her boyfriend for information on contraceptives (in turn he was advised by his mother, although he did not always take her advice) and another expected her boyfriend to buy condoms. In contrast, some young women had never discussed contraceptive use with their partner. Whilst unmarried men in Hubei perceived that the responsibility should ideally be a joint one, they reported that men were more likely to be the main decision-makers regarding contraceptive use, especially in relation to condom use.

*“He (partner) discussed contraceptives with me, but I didn’t know much about it”.*

(FGD with unmarried women, Hubei, medium GDP context).

#### Partner involvement in PAFP is desired but not always implemented

When asked about their partner’s involvement during the abortion process, most women were keen to have partners accompany them to the facility for at least part of the process. A number of female participants across the three provinces said that their partners had accompanied them for their recent abortion and most male service users had accompanied their partners at some stage during the procedure. Inconsistent rules and procedures for partners wishing to be involved were reported across different institutions; however men were often able to wait with their partners and in some cases were invited to join them after the abortion and while information on FP was provided.

The majority of women across all three provinces felt that their partners should be involved in the provision of PAFP services. This view was also mirrored by the majority of male partners interviewed in all three provinces, although one mentioned that he felt women were in a better position to make any final decisions. A number of these men and women stated that it is important for men to be involved in these services in order for them to understand the situation of women with more sensitivity and to be able to jointly take responsibility for contraception. One male partner in Yunnan connected this to avoiding subsequent abortions.

*I do think it’s important to involve my husband. If he is there with me, he will know more about contraception and he will care more about me.*

(IDI with married woman user, Zhejiang, high GDP context).

### Relationships with health providers: Limited and directive counselling in experiences of PAFP

#### Provision of FP counselling focused on information provision

Just under half of service users or their partners interviewed reported receiving no information about FP before, during or following the abortion, with some variation between provinces. For instance, in Zhejiang most had not received any specific information about contraceptive methods following their abortion, although one mentioned that she had been given some very basic advice. In contrast, many of the service users interviewed in Hubei had received some information about contraceptive methods from providers.

Where users did receive FP information, it appeared that the focus was often on limited to several methods. For example, in Yunnan female participants were mainly given information one or two types of contraceptive method, usually either IUDs, implants or condoms but, in some cases, OCs and female sterilisation. One service user reported simply being told to use FP, with no further details provided.

*Doctors told me that I have to use contraception at least 6 months before next pregnancy. They didn’t counsel me how to use contraception. They just told me to use condoms and said that pills have a lot of side effects. They mentioned ‘safe period’ is not safe. If my husband uses condom, he has to use it every intercourse. It took about 2–3 min.*

(IDI with married woman service user, Zhejiang Province, high GDP context).

Many service users were sometimes unable to remember what they had been told in any detail, especially once they had decided on a method:

*I did not remember so much (information). I know condom suits me. [She] told me not to use pills. I remembered this….*

(IDI with unmarried woman service user, Hubei province, medium GDP context).

Only one service user (from Yunnan) explicitly described the experience of counselling as positive and felt the advice she received had increased her knowledge about contraception.

Some participants (around a third of the sample) were able to use the information they received on contraceptives during post-abortion counselling. Some of these participants either switched from short-acting methods to long-acting methods (usually having an IUD inserted), or reported that they began using short-term methods more effectively (primarily using condoms consistently). Others who had not been consistently using modern contraception decided to use it following (usually brief) post-abortion counselling. One married male partner from Hubei said that he had realised through the information given that the ‘safe period’ method was not effective and that IUD would be better; he was currently using condoms with his wife and they planned IUD insertion in the future. The following vignette constructed from an interview illustrates another case of planned behavior change in response to post-abortion counselling advice:

*Wang is an unmarried woman living in Hubei province. Wang has usually relied on condoms and emergency contraception (EC), which she purchased from a pharmacy. EC was her main contraceptive method, which she used more than three times a year and felt was very convenient. She did not discuss contraception methods with her partner or colleagues and she did not seek or receive advice from any doctors. Most of her contraceptive knowledge was from TV adverts. Wang decided to terminate her last unintended pregnancy because she was not married. Service providers gave her and her partner some contraceptive information after the abortion and advised her not to rely on EC. Although she was asked by doctors whether she had any questions, Wang did not know what or how to ask. However she thought the doctors were very professional and their advice was helpful. She now uses condoms since she worries that EC might have adverse effects on her future pregnancy so she has decided not to use it. After child birth, she would like to use IUD because it is a long-term method. She regretted not knowing much about contraceptives before the abortion.*

The vignette illustrates that information provided in post-abortion counselling about the disadvantages of EC as a regular contraceptive method was instrumental in encouraging Wang towards more consistent use of condoms. However, it is unclear whether she now understands fully how to use condoms effectively, or is able to negotiate them with her partner, since there was no dialogue on these issues with a health provider.

#### Relationship with providers: Perceived authority but two-way communication is limited

Both women and men agreed that doctors were the most reliable sources of FP information:

*The most reliable (information) is what doctors said. It is more authoritative.*

(IDI with unmarried young woman, Hubei Province, medium GDP context).

However, they were perceived to be too busy to provide FP counselling in any depth.

*Doctors are very busy in the hospitals. They are not able to spend lot of time to communicate with you.*

(IDI with woman service user, Zhejiang Province, high GDP context).

According to users’ narratives, where providers (usually referred to as doctors) did conduct FP counselling, few encouraged questions or attempted to discuss the advantages and disadvantages of different contraceptive methods in their patients’ specific situation. Users also felt constrained in asking questions, even when they did not fully understand the information given. For example, one married male partner in Hubei province said that he felt embarrassed to ask questions. Another felt that contraceptive information provided by doctors was too technical and difficult to understand.

#### Limited counselling could not address underlying constraints on effective contraceptive use

A number of participants expressed uncertainty about their future contraceptive plans following limited counselling, often because the underlying reasons for contraceptive failure or non-use had not been discussed openly or resolved. For example,

*Zhou is a married woman living in Hubei province. She had used IUD before childbirth, and resumed this once her child was about one year old, despite experiencing prolonged menstrual bleeding. However, several years later, she had an ectopic pregnancy with an IUD* in situ*. She did not discuss contraception with her husband and said she can decide what method to use by herself. After the ectopic pregnancy, she had her IUD removed. Since then, although the couple sometimes used condoms, her husband was reluctant to do this, so they used the rhythm method as their main form of contraception and had used EC several times. At this point she became pregnant and had an abortion. She received limited post-abortion FP counselling during which some contraceptive methods were introduced, including IUD and contraceptive injections, but no further information was given about their advantages and disadvantages. She didn’t ask the doctor any questions. The provider recommended IUD to Zhou because she did not want to use oral contraceptives. However, Zhou did not want to use the IUD, because of to her experience of ectopic pregnancy. She felt oral contraceptives were unsafe due to side effects. One of her female friends used the ‘Sino-implant’ but experienced heavy bleeding, which led to her developing anaemia. Zhou therefore continues to use the ‘rhythm method’, despite being aware of its relative ineffectiveness, since she perceives her choices as very limited and sees her risk of pregnancy as low because she is over 40.*

Zhou’s case illustrates both the limited choices available within contraceptive provision and social norms and also the limitations of brief contraceptive counselling to address her dilemma, since she was recommended a method that she did not feel comfortable with but did not feel able to discuss this with the provider. Another case illustrates profound ambiguities about future contraceptive plans in a context of limited counselling:

*Chen is a married ethnic minority womanliving in Yunnan who had an abortion six months ago. After her first child was born, she had an IUD inserted. She and her husband chose the IUD because they thought it was a long-acting method and Chen was happy with the option. However the second month after the IUD was inserted, she became pregnant. She had used condoms before the IUD insertion and continues to use them now following the abortion, although she thinks that condoms are little bit uncomfortable. She is aware of other forms of contraception, such as oral contraceptives but she feels that they are bad for her health as she may gain weight or experience acne, according to peers. After the abortion, the doctor did not provide much contraception information and just said that the IUD is ‘not suitable’ for her and that condoms may be better for them. Since then she uses condoms, and doesn’t plan to change in the future. However, when asked if Chen uses condoms every time they have sex, Chen explained that when her husband is drunk he won’t use them* ***.***

Chen’s case again reveals the limited options available within health service provision and social norms and the limitations of brief counselling in ensuring that they use contraception effectively: the couple are not fully comfortable with the new method and have neither been counselled in using it effectively nor on other available options.

## Discussion

### Social norms and networks influencing contraceptive use need further attention

Our results provide important insights into the perceptions and experiences of women undergoing unintended pregnancy and subsequent abortion, their contraceptive decision-making, and missed opportunities in the delivery of post abortion family planning (PAFP) to appropriately address their FP needs. Interviews revealed participants’ perceptions that changing social norms of pre-marital sexual activity, are linked to increased numbers of abortions. Available evidence suggests that young, unmarried and migrant women are disproportionately represented among women seeking abortions [8, 18]. Our study elucidated some of the social mechanisms underpinning this evidence in the Chinese context. According to our study, social norms and perceptions about suitability and safety, as well as gendered norms about decision-making on contraceptive methods contribute to greater challenges for these groups of women. For example, in line with a number of studies internationally, long-acting methods may be perceived as appropriate only after child-bearing (within marriage in the Chinese context), and young women often rely on their partner for information about contraceptives and/or willingness to purchase and use condoms. Participants perceived that stigma was a barrier to young, unmarried women’s and men’s ability to negotiate FP information and services [24]. The need to develop more inclusive, ‘youth-friendly’ services to address stigma, among other barriers has been well established internationally [25, 26]. In China, research has shown that work also needs to be done with parents and other ‘gatekeepers’ to address ambivalent and/or negative attitudes towards improving sexual and reproductive health education among young people [27, 28].

### Anxieties about contraception are exacerbated by lack of information and person-centred support

International literature suggests that concerns about side effects and health risks are a common reason for non-use of contraceptive methods by sexually active couples who do not intend a pregnancy: analyses of Demographic and Health Surveys (DHS) in 51 and 35 low and middle income countries respectively found that this reason accounted for non-use for between 23 and 35% of married women [22] and 37.3% of sexually active women aged 15–49 [29]. Similarly a systematic review of the evidence from ‘developed’ countries regarding desirable and undesirable qualities of long-acting reversible contraception (LARC) identified concern about side effects as a common perceived negative quality of LARC [30], whilst qualitative research identified misconceptions about IUDs and other LARC as a factor in low utilisation among young women in Australia [31] Our data from women of varying ages and backgrounds suggest that relatively high levels of anxiety about contraceptive methods are a contributing factor in China, including widespread concerns about side-effects (particularly from oral contraceptives, but also IUDs), effects on future fertility, and reliability. The context for this is very low availability of person-centred counselling on contraceptive methods that explains the relative advantages and disadvantages of a range of modern methods in the specific situation of the individual client. For example, the general orientation of FP counselling towards encouraging IUD use can mean that those women who have experienced side effects or difficulties with IUDs are not enabled to identify an alternative method that meets their needs through FP counselling. Moreover, clients who have experienced the failure of their existing method (for example pregnancies with IUD in situ, or, more often, related to inconsistent use of a method) are generally not facilitated to reflect on this and the implications for future contraceptive use through existing counselling. For young unmarried people, reliable and easily accessible sources of information and options for modern methods are even more limited. The combination of limited information and choices with anxieties about side effects also underlies the widespread use of unreliable methods, such as the ‘rhythm’ method, and the regular use of emergency contraceptives. Our data also revealed that commercialisation of reproductive technologies influenced participants’ perceptions of product quality, potentially undermining local and public provision of FP services such as distribution of free condoms.

### Consideration of gendered power dynamics in mediating contraceptive use is needed

Our findings revealed the significant influence of gendered power relations on contraceptive use and non-use. Limited evidence from China suggests that while attitudes have become more accepting of sexual permissiveness for unmarried people, women remain at a disadvantage in terms of social norms guiding such behaviour [32, 33]. For example, a study exploring Beijing women’s experience of coerced sex and virginity loss highlighted how the cultural shift in attitudes towards sex may leave some women in a very precarious position, given the pressure they feel from society to act as ‘traditional’ women and the pressure from their partners and boyfriends to be sexually available. A number of the women in this study were forced to have abortions following coercive sexual encounters [33]. Our findings show how unequal gender relations impact not only on the sexual encounter itself, but on whether and what type of contraception is used. While there was general consensus in our data that women and men should make joint decisions about contraceptive use, there were a number of examples illustrating women’s relative lack of power to use a method they preferred, or to use contraception at all; for example where women reported that their partners sometimes refused to use condoms. Evidence from other contexts demonstrates how gendered power dynamics impact on contraceptive use. For example, recent data collected in Sub-Saharan Africa revealed covert use of contraceptive methods due to male resistance, perceived or otherwise, to contraceptive use [34]. Analyses of DHS data from 35 and 51 low and middle income countries respectively found that partner opposition was given as a reason for non-use of contraception by 22.4% of sexually active women who did not desire a pregnancy [29] and between 11 and 27% of married women who did not desire a pregnancy [22]. Increasingly, interventions aim to engage men in reproductive decision-making based on evidence that joint decision-making is more likely to promote contraceptive use among couples [35, 36]. Our study suggests that it is important to involve male partners in PAFP in China and to facilitate reflection on communication about contraception within post-abortion counselling.

### Comprehensive counselling is a key component in post-abortion family planning

Finally, related to the existing provision of counselling, our study illustrates a range of limitations for effective communication between service users and service providers about their FP experiences and future options. While studies have shown that low levels of SRH knowledge affects vulnerable groups in particular [19–21], our findings demonstrate how communication barriers with providers related to FP knowledge and decision-making affect women of all ages and backgrounds. Many participants received some information regarding contraceptive methods, and some reported being able to use this information to adopt more effective contraceptive behaviour. However, only one woman explicitly described having experienced a discussion that she perceived as increasing her general knowledge about contraception. The lack of in-depth person-centred counselling also meant that patients with facing medical and/or social challenges to using a method they found acceptable did not receive support in considering how to deal with these challenges. Women who had experienced complications with more ‘accepted’ types of contraception, such as IUD, were placed in a particularly difficult position, with little opportunity to discuss their specific needs. Furthermore, even where participants reported being able to apply the information they received, they rarely described receiving in depth counselling about these decisions, for example, to discuss ways in which they could improve their correct and consistent use of condoms. WHO guidelines emphasise the importance of informed decision-making in the provision of contraceptive information, advice and services that respect human rights. Among other aspects, health workers should provide non-directive and comprehensive counselling that allows clients to make autonomous decisions about their contraceptive use, without coercion or misinformation [37]. Despite the existence of such guidelines, research on post-abortion contraceptive services demonstrates the challenges of conveying information about contraceptive methods in a sensitive manner at a time that is already difficult for women (and their partners). A study in the UK found that some health workers feel they must guide women towards a particular ‘choice’ of contraceptive method [38] while a study in Nepal found that health workers made judgments about the suitability of certain methods for certain groups of women, therefore biasing clients’ ability to make informed and autonomous decisions [39]. Addressing such challenges in China will require a shift in attitudes and skills of providers as well as the allocation of resources to enable them to spend sufficient time with clients [40].

## Conclusions

In conclusion, our study conducted in three Chinese provinces revealed a number of important findings regarding perceptions and experiences of contraceptive use, abortion and post-abortion family planning (PAFP) services. Social networks and the norms that underpin them play a key role in mediating attitudes and behaviour towards abortion and contraception in China. Linked to this, commercial pressures are also relevant in promoting specific types of methods. Access to non-commercial, reliable information on contraceptive methods, their efficacy and potential side-effects, to support that given by providers is needed, and may be most accessible for young people through internet and/or social media provision. Although respect for the authority of providers can offer impetus for individual behaviour change, it can also create social distance which reduces opportunities for clients to discuss their difficulties regarding contraceptive use, ask questions or make autonomous decisions on a suitable method; this is particularly, but not exclusively, the case for young, unmarried clients. In light of the influence of gendered power relations, more sensitive probing may be required during counselling and, where women choose this, efforts should be made to include their male partners in the process. Taking these relational influences into account, PAFP services must go beyond simple information provision to ensure that providers take a more person-centred approach that considers the most appropriate method for individual clients and probes for the underlying influences on contraceptive (non)use. Providers face challenges to this approach related to the time they have available to see clients and the current lack of training in person-centred approaches. Sufficient numbers of providers trained in person-centred approaches are therefore needed. Specific attention to the positionality and skills of providers for counselling younger clients may be needed. Further research is also required to understand better the needs of young unmarried migrants who experience additional risks related to their ‘floating’ residence status.

## Abbreviations

DHS: Demographic and Health Survey
EC: European Commission
FGD: Focus Group Discussion
FP: Family Planning
FP7: the Seventh Framework Programme
GDP: Gross Domestic Product
IDI: In-Depth Interview
INPAC: Integrating post-abortion family planning services into existing abortion services in hospital settings in China
IUD: Intra-Uterine Device
OC: Oral Contraceptive
PAFP: Post-Abortion Family Planning
WHO: World Health Organisation

## Footnotes

1
**In**tegrating **p**ost-abortion family planning services into existing **a**bortion services in hospital settings in **C**hina (INPAC) is funded by European Commission (EC) under the Seventh Framework Programme (FP7). Project number: 282,490.

2 The Chinese word used was “生活空虚”, which translates as feelings of emptiness and depression.

3 The Chinese expression was,“放环就是老一辈传统下来的东西,根本没多想”, which carries as sense of accepting a practice of previous generations without any further consideration
